# Use of tissue adhesive for neonatal intravenous access devices: A scoping review

**DOI:** 10.1007/s00431-024-05800-3

**Published:** 2024-10-05

**Authors:** Sabrina de Souza, Mari Takashima, Thiago Lopes Silva, Linda Nugyen, Tricia M. Kleidon, Luke Jardine, Tim R. Dargaville, Amanda Ullman, Deanne August, Patricia Kuerten Rocha

**Affiliations:** 1 The University of Queensland, Brisbane, QLD Australia; 2 Universidade Federal de Santa Catarina, Florianopolis, SC Brazil; 3 Children’s Health Queensland Hospital and Health Service, Brisbane, QLD Australia; 4 Mater Clinic Unit, Brisbane, QLD Australia; 5 Queensland University of Technology, Brisbane, QLD Australia

**Keywords:** Neonatology, Vascular access devices, Peripheral intravenous catheter, Central venous catheters, Tissue adhesive, Securement device

## Abstract

Neonates often require vascular access devices for medication or fluid therapy, but a third of devices fail before treatment completion or end with a complication. For adults and children, securing these devices with tissue adhesive (TA) increases the dwell and reduces complications. However, there is a lack of evidence for the neonatal population. This scoping review aimed to assess the evidence of TA for vascular access devices in neonates. The Arksey and O’Malley’s (2005) framework was used. The inclusion criteria covered studies published from 2007 (when TA was first reported for use in vascular access devices) to June 2024, available in English, Portuguese, and Spanish, across six databases. Two independent reviewers assessed the studies using Covidence software, with a third reviewer resolving conflicts. Quality assessment was performed using the Mixed Methods Appraisal Tool. From 981 identified studies, 12 were included. Most studies (*n* = 5, 41.7%) enrolled between 100 and 500 neonates with vascular access devices. Publications originated from four regions and were observational studies (*n* = 6, 50%), quasi-experimental (*n* = 3, 25%), and case series (*n* = 2, 16.7%) with one randomized controlled trial (8.3%) focusing on umbilical venous catheters (UVC). The most common TA composition used was a combination of *n*-butyl- and 2-octyl- cyanoacrylate (*n* = 4, 33.3%). The amount of TA applied varied across studies, and often TA was part of a bundle (*n* = 7, 58.3%). Most studies applied TA to central venous access devices (*n* = 10, 83.3%) and 2 (16.7%) in peripheral devices. Although there was variation in device failure, the studies generally indicated a reduction in complications such as dislodgment (central catheter: 11.3% [peripherally inserted central catheter {PICC}] to 24.6% [UVC] in non-TA group vs 0.7% [PICC] to 7.7% [UVC] in TA group), device-associated bloodstream infections (central: 7.7% [UVC] and incidence of 2.76/1000 catheter days [PICC] in non-TA group vs 3.1% [UVC] and incidence of 0.99/1000 catheter day [PICC] in TA group), and phlebitis (13% in non-TA group vs 3% in TA-group), as well as increased dwell time in peripheral catheters. Most studies included both term and preterm neonates but did not differentiate between them in their analyses. Skin assessment, life of first dressing, and follow-up of catheters and patients were not reported in most studies.

*Conclusion*: TA may reduce complications in vascular access devices, but the evidence in neonates is limited and varied. Many studies include TA as part of bundle, making it difficult to isolate its effects. Additionally, the current evidence lacks robustness due to the design limitations of the studies. Future research should focus on randomized controlled trials to evaluate TA’s effectiveness and safety in preventing device failures and complications in neonates, considering different subgroups, to ensure the safety of TA in these nuanced populations.

**Table:** 

**What is Known:** *• Research in adults and pediatrics provides evidence supporting the use of tissue adhesive (TA) for vascular access devices, showing a positive impact in reducing failures and complications.* *• The use of TA in neonates needs to be carefully considered due to their unique characteristics.* **What is New:** *• There is a gap in the literature on the use of TA for securing vascular access devices in neonates, particularly regarding its safety and effectiveness in preventing failures and complications.* *• Further studies are needed to provide robust evidence verifying the effectiveness and safety of TA in this population.*

**Supplementary Information:**

The online version contains supplementary material available at 10.1007/s00431-024-05800-3.

## Introduction

Almost every neonate admitted to a neonatal unit requires an intravenous device for medications, fluids, nutrition, or blood infusion therapies [1]. Despite the frequent utilization of either central venous access devices (CVADs) or peripheral intravenous devices, failures and complications occur for over a third of devices [2–4]. These complications not only harm this fragile population but often necessitate an additional insertion procedure, which has implications on many levels, including neurodevelopment, pain, and additional handling [5]. The unique characteristics of neonatal tissues near birth, i.e., immature immune response [6], extracellular fluid in surrounding space, and poor functionality of preterm skin [7, 8], may contribute to higher rates of infection, especially for those of lower gestational age [6]. Additionally, these complications can affect the future health of the vein network, leading to lifelong deleterious sequelae [5, 9].

One innovation for vascular access practice is the use of tissue adhesive (TA) (a medical grade liquid glue/adhesive), for vascular access device securement [10]. Since the development and reporting in adult literature in 2007 [11, 12], TA has been improved, with two generations and distinct characteristics [13]. Compositions of either *n*-butyl-cyanoacrylate (NBCA) or the 2-octyl-cyanoacrylate (OCA) [13], which lead to other derivatives, such as NBCA + softener, and NBCA + OCA, are available from different manufacturers, with different properties [14]. When used with vascular access devices, the TA is intended to “seal” the insertion site to prevent post-insertion ooze, micro-motion, and the entry of microorganisms; moreover, the bacteriostatic property and high tensile strength have been linked to reducing catheter associated-bloodstream infection (CLABSI) and dislodgment respectively in adults [15, 16].

Evidence from a systematic review has established the effectiveness of TA in adults and pediatric populations [3, 17–22]; however, the evidence and recommendations for neonates remain sparse. In addition, the well-consolidated evidence from generalized older populations does not extend to neonates. Neonates are uniquely vulnerable due to their different physiological characteristics (e.g., immature vasculature and delicate skin), treatment, and population-specific complication rates, experiencing the highest rates of vascular access device-related complications and failures [2, 4, 23–25]. Therefore, the aim of this review is to scope the use of TA for vascular access devices in neonates by the following:

i) Describing the use of TA in the studies
ii) Exploring the characteristics of neonates’ included in the study sample
iii) Describing the type of vascular access device secured with TA
iv) Exploring failures rates of vascular access devices secured with TA
v) Reporting complications rates of the vascular access devices secured with TA (e.g., partial or total dislodgment, thrombosis, occlusion, infiltration/extravasation, infection related, catheter breakage, pain)
vi) Reporting skin assessment, dwell time of the vascular access devices, follow-up of catheters and patients, life of the first dressing

## Methods

### Design

**Fig. 1:**
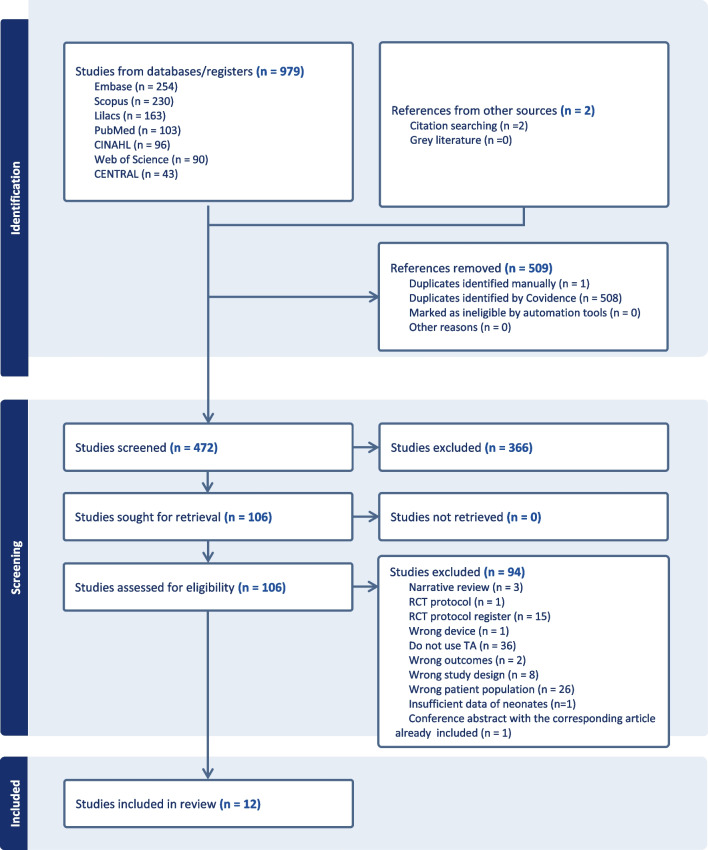
PRISMA-ScR flow diagram extracted from Covidence

This scoping review followed the Arksey and O’Malley’s [26] methodological framework and is reported according to the Preferred Reporting Items for Systematic Reviews and Meta-Analyses extension for Scoping Reviews (PRISMA-ScR) (Fig. 1) [27].

### Search strategy

A comprehensive and systematic electronic search was developed in collaboration with a Health Science Librarian (D.H.) from the University of Queensland. The strategy used relevant keywords and MeSH terms, adjusted for database-specific headings, focusing on population, catheter device, and TA. Searches were conducted in EMBASE, CINAHL (Ebsco), PubMed, Web of Science, Cochrane Library (CENTRAL), Scopus, and Latin American and Caribbean Health Sciences Literature (LILACS). ClinicalTrials.gov, the Australian New Zealand Clinical Trials Registry, and the International Clinical Trials Registry Platform were manually searched for additional RCT protocol articles. Reference lists of selected articles were also reviewed for further studies (Supplementary Table 1).

The literature search was conducted from May to June 2024 (up to June 18). Two review authors (S.S. and M.T) independently screened titles and abstracts before full-text review using Covidence (Veritas Health Innovation, 2023), with disagreements resolved by a third reviewer (D.A) (Fig. 1). For articles in Portuguese or Spanish, initial screening was planned by different reviewers (T.L.S. and S.S.), with a third reviewer (P.K.R.) resolving disagreements.

### Inclusion and exclusion criteria

The population included was neonates with TA securing their vascular access devices, including CVADs, such as peripherally inserted central catheter (PICCs), umbilical venous catheter (UVC), tunneled cuffed, and non-tunneled central venous catheters, centrally inserted central catheter (CICC); femorally inserted central catheter (FICC); and peripheral catheter devices, such as midline catheters and peripheral intravenous catheter (PIVC). Neonates were defined as any live-born infant receiving care in a hospital or healthcare setting until discharge from a neonatal facility or unit or receiving care in a specialized nursery setting [8]. Publications reporting on mixed populations, such as pediatrics including neonates, were included if neonatal subset data were available. The review covers studies from Neonatal Unit Services worldwide using TA in vascular access devices, published between 2007 and 2024. This period is notable as the first study on TA for vascular access devices appeared in 2007. Included study designs were RCTs, pilot RCTs, non-randomized studies, cohort studies (prospective and retrospective), quasi-experimental, case–control, observational studies, and case series. Only studies in English, Portuguese, and Spanish were assessed. Excluded were case studies, protocols, conference abstracts without extractable data, editorials, narrative reviews, and theses.

### Data extraction

Data were collated using a standardized form, which included details such as study authors, year of publication, country, study design, type of center, population, total neonates studied, type of vascular access device studied, total number of catheters studied (those that received TA or were compared to non-TA use), types of TA used (including composition), quantity (in ml or drops) and timing of TA application, neonate classification (e.g., low-weight, preterm, age), vascular access device failure and complications, catheter dwell time, skin assessment, follow-up of catheters and patients, and life of first dressing. When TA was reported as part of a bundle, the components of the bundle, as well as the bundle or TA outcomes, were documented according to the study assessed.

### Quality appraisal

Empirical studies were evaluated for risk of bias using the Mixed Methods Appraisal Tool (MMAT) [28] (Supplementary Table 2). This appraisal tool utilizes a checklist for study methodological quality against five criteria [28]. Each study was assessed by S.S. and L.N., and M.T and D.A. reviewed disagreements.

## Results

The report of study screening, inclusion and data extraction is presented in a PRISMA flow diagram [27] (Fig. 1). Of the 981 studies reviewed, 472 were screened after duplicates removed. Of these, 12 met the criteria and were included in this review.

### Population and vascular access device characteristics

Most of the studies enrolled between 100 and 500 neonates (*n* = 5, 41.7%), and 100 to 500 vascular access devices (*n* = 5, 41.7%) performed in a combined neonatal population (full terms and pre-terms) (*n* = 11, 91.7%). In most of the studies, TA was used for CVADs (*n* = 10, 83.3%) (Table 1). The majority of them were conducted in Italy (*n* = 7, 58.3%) and had an observational study design of a retrospective nature (*n* = 4, 33.3%) with only one (8.3%) RCT published (Table 1). In addition, fewer studies (*n* = 5, 41.7%) evaluated outcomes specifically related to TA, while the majority (*n* = 7, 58.3%) focused on bundled interventions that included TA.

**Table 1:** Selected study description

Study characteristics	*N* (%)	Study reference
Participants description		
Number of participants		
Number of neonates		
< 100	4 (33.3%)	[29–32]
100–500	5 (41.7%)	[33–37]
500–2000	1 (8.3%)	[38]
> 2000	2 (16.7%)	[24, 39]
Neonates’ classification		
All (terms and preterm)	11 (91.7%)	[24, 30–39]
Preterm, with weight < 1500 g	1 (8.3%)	[29]
Vascular access device description		
Number of vascular access device		
< 100	4 (33.3%)	[29–32]
100–500	5 (41.7%)	[33–37]
500–2000	1 (8.3%)	[38]
> 2000	2 (16.7%)	[24, 39]
Type of vascular access device		
CVAD		
PICC/CICC/FICC	9 (75.0%)	[29–36, 38]
UVC	1 (8.3%)	[37]
PIVC	2 (16.7%)	[24, 39]
Study description		
Country of origin		
Belgium	2 (16.7%)	[33, 35]
Italy	7 (58.3%)	[29–32, 34, 36, 37]
Qatar	3 (25.0%)	[24, 38, 39]
Study design		
Randomized controlled trial	1 (8.3%)	[37]
Quasi-experimental studies		
Pre and post-intervention	3 (25.0%)	[33, 35, 36]
Observational studies		
Prospective study	2 (16.7%)	[31, 34]
Retrospective study	4 (33.3%)	[24, 30, 38, 39]
Case series	2 (16.7%)	[29, 32]
Single or multisite study		
Single site	11 (91.7%)	[24, 30–39]
Multisite	1 (8.3%)	[29]
Year of the study publication		
2021	3 (25.0%)	[24, 29, 33]
2022	4 (33.3%)	[30, 32, 36, 38]
2023	4 (33.3%)	[31, 35, 37, 39]
2024	1 (8.3%)	[34]
Assessment of the outcomes		
TA assessment	5 (41.7%)	[24, 36–39]
Bundle assessment	7 (58.3%)	[29–35]
TA description		
TA composition		
Pure *n*-butyl	2 (16.7%)	[29, 36]
Pure 2-octyl	1 (8.3%)	[35]
Combination (*n*-butyl + 2-octyl)	4 (33.3%)	[30, 37–39]
Pure *n*-butyl or combination	2 (16.7%)	[31, 34]
Not reported	3 (25.0%)	[24, 32, 33]
Quantity of application*		
“A drop”^α^	1 (8.3%)	[33]
“Few drops”^α^	1 (8.3%)	[36]
Four drops^α,£^	2 (16.7%)	[34, 37]
Not reported	8 (66.7%)	[24, 29–32, 35, 38, 39]
The moment that the TA is applied:		
In the insertion procedure/after insert the catheter	10 (83.3%)	[24, 29–37]
In the insertion and in every dressing change	1 (8.3%)	[39]
Not reported	1 (8.3%)	[38]

^α^TA applied in PICC/CICC/FICC

^£^TA applied in UVC

*TA* tissue adhesive, *PIVC* peripheral intravenous catheter, *CVAD* central venous access device, *PICC* peripherally inserted central catheter, *CICC* centrally inserted central catheter, *FICC* femorally inserted central catheter

Studies included were conducted as early as 2021 (*n* = 3, 25%). The composition of the TA varied, with the combination of 2-octyl- and *n*-butyl-cyanoacrylate being the most reported (*n* = 4, 33.3%). Most studies did not specify the amount of TA applied (*n* = 8, 66.7%), but when provided, the most common amount was 4 drops (*n* = 2, 16.7%), primarily applied during the insertion procedure, after catheter placement (*n* = 10, 83.3%), with only one study (8.3%) reporting its use during dressing changes (Table 1). The description of the TA use and outcomes can be found in Supplementary Table S3.

### Studies designs

#### TA only

TA was specifically the focus of five (41.7%) studies. One retrospective study demonstrated a significant reduction in catheter failure when comparing TA for PICC to the control group (*n* = 259, 27.9% vs *n* = 91, 11.7%; *p* < 0.001) [38]. However, a non-blinded single-center RCT for UVCs did not identify a reduction in failure (*n* = 3, 4.6% vs *n* = 4, 6.2%) [37]. For PIVCs, one observational study reported significant differences for device failure between the TA and non-TA groups [39] (Supplementary Table S3).

All studies reported a reduction in at least one complication, though the specific types of complications varied. The RCT for UVCs demonstrated a significant reduction in dislodgment (*n* = 16, 24.6% vs *n* = 5, 7.7%, *p* = 0.016) and a non-significant reduction in CLABSI (*n* = 5, 7.7% vs *n* = 2, 3.1%) [37]. An observational study of PICCs reported a significant decrease in CLABSI incidence rates (2.76/1000 days vs 0.99/1000 days, *p* < 0.001) [38]. In a PIVC study, the complication phlebitis (*n* = 594, 13% vs *n* = 123, 3%) had the most proportional difference comparing non-TA and TA groups [39]. Dwell time for PIVCs with TA was improved in both studies (31 ± 25 and 37.1 ± 31.1 h) respectively compared to non-TA group (28 ± 18 and 31 ± 24.3 h) [24, 39], while dwell time of PICCs remained unaffected [36, 38] (Supplementary Table S3).

#### Bundle studies

TA was incorporated into a practice bundle for seven (58.3%) studies, all involving CVADs. Of these, only one studies assessed device failure [30]. Among the studies evaluating complications, two demonstrated a significant reduction in CLABSI after including TA in the bundle [33, 35]. Additionally, dislodgment decreased significantly in two studies, although these studies did not show a difference in thrombosis [33, 35]. Conversely, occlusion did not reduce in one study [35] but was reported as significantly reduced in another study that grouped it with breakage and disconnection [33]. Dwell times were only reported for CICC and varied widely, ranging from 10.88 ± 6.41 days to 39 ± 25 days [30, 32] (Supplementary Table S3).

#### Neonates’ classification and outcome reports

Most studies were conducted with term and preterm neonates, while one case series study [29] specifically evaluated TA in the CICC bundle for preterm neonates weighing below 1500 g and reported no complications. Most studies do not differentiate between term and preterm neonates in their analyses. Nevertheless, three studies provide separate analyses [33, 37, 38]. For instance, an observational study found higher rates of suspected sepsis and death due to CLABSI in neonates of 23–27 weeks (suspected sepsis *n* = 67, 11.6%; death due to CLABSI *n* = 8, 1.4%) compared to 28–31 weeks (suspected sepsis *n* = 35, 4.3%; death due to CLABSI *n* = 0) and others gestational ages [38]. Similarly, a quasi-experimental study [33] found that 88.9% (16/18 cases) of CLABSIs affected patients < 32 weeks. The study noted a significant reduction in CLABSIs after TA implementation in a bundle, decreasing from 16 to 1 case (*p* = 0.04) (Supplementary Table S3).

### Skin assessment, life of dressing change, and follow-up

Most studies (*n* = 8, 66.7%) did not assess skin integrity related to TA use [24, 29, 30, 33, 34, 36, 38, 39]. Among those that did (*n* = 4, 33.3%), none identified any cases of skin complications [31, 35, 37], although one study reported a skin ulcer, caused by subcutaneous anchor system in one (1.4%) patient [32]. Moreover, the life of first dressing was not assessed in majority studies. Only one study noted that no dressings needed to be changed due to bleeding at the insertion point attributing this to the use of TA (pure 2-octyl) as a hemostatic agent [35].

Follow-up of the catheters and patients was reported in three (25%) studies [31, 34, 37]; an RCT comparing UVC securement performed ultrasound 1 week after catheter removal, and two studies with CVADs included follow-up 2 weeks post-insertion [31, 34]. The remaining studies (*n* = 9, 75%) did not report follow-up regarding catheter completion, failure, or treatment [24, 29, 30, 32, 33, 35, 36, 38, 39], and none tracked individual neonates for future outcomes such as length of stay or discharge [24, 29–36, 38, 39].

### MMAT (Supplementary Table S2*)*

Quality assessment using the MMAT [28] found that all studies included clearly reported the research question. Eleven (91.7%) of the studies that collected data effectively addressed the research question, while one (8.3%) provided an unclear report [32]. Regarding other MMAT criteria, only one (8.3%) study [38] met all criteria for the corresponding categories to be considered at low risk of bias. The remaining studies (*n* = 11, 91.7%) had at least one MMAT criterion unmet. For example, one study had missing endpoints for some catheters and unclear reporting, while [36] another study [39] reported the implementation of the TA but used IvWatch® concurrently, and this potential confounder was neither reported nor assessed [39]. Moreover, there was an insufficient reporting about TA usage [24], outcome definition, especially safety [30], and skin integrity [38]. Furthermore, while clinical outcomes were assessed in the studies evaluating the bundle, there was a lack of evaluation of individual bundle elements, implementation outcomes, or compliance measures [33, 34].

## Discussion

Vascular access devices, including central or peripheral catheters, pose a high risk of failure and complications, especially in fragile populations, such as neonates [4]. TA has emerged as a promising approach to enhance device security and improve outcomes. However, the unique physiological characteristics of neonates necessitate careful evaluation of TA’s safety and efficacy [2, 4, 23, 25]. This concern is particularly emphasized for preterm and extremely preterm infants, who have immature skin more prone to damage [23, 40], have high risk for absorption toxicity [41], and are at higher risk for infections due to their underdeveloped immune systems [6]. This scoping review revealed a limited body of evidence specific to TA use in neonates. There is considerable variation in the quality and design of the studies, with most incorporating TA as part of a bundled intervention, making it difficult to isolate its effects. This underscores the need for caution when implementing this practice in such a highly specific and fragile population without robust evidence. It also highlights the necessity of a future RCT focused on this intervention to address the gap in this population.

A key finding was the lack of standardization in TA composition and dosage for neonates. The different types of TA composition were not compared in the studies included in this review or in other populations. Moreover, the study designs in this review do not allow for such comparisons. While manufacturers typically recommend 2 drops of TA for general use [15, 42], they do not provide specific guidance for neonates, including considerations for different vascular access devices and body surface area [43]. This review observed variability in practice, with inconsistent reporting of dosage and safety outcomes. A pilot RCT in pediatric PICCs found that two drops of TA at insertion are sufficient [44], and manufacturers warn that excess TA pooling can cause heat, pain, or discomfort [15], which is particularly concerning for a non-verbal neonates with heightened pain responses [5]. This variability and lack of clarity underscore the urgent need for well-designed RCTs to evaluate the effectiveness and safety of various TA formulations, ensuring precise dosing tailored to specific devices and neonatal characteristics.

The safety profile of TA for neonates remains unclear. Although some studies reported no skin injuries, the evidence is limited due to study design and lack of follow-up. This uncertainty is concerning, as a systematic review in adults and pediatrics found TA increases the risk of skin irritation or damage compared to other securements and standard care (RR 1.64, 95% CI 1.10–2.44) [10]. The special conditions of preterm infants, such as skin immaturity and altered sensory responses, make this subgroup significantly more vulnerable than other neonates and necessitate special attention for interventions [23, 45]. The diverse neonatal population, from extremely preterm to full-term infants, requires careful assessment for TA safety. Most studies did not stratify outcomes by gestational age, and data on age at TA application were limited. The few that did [33, 37, 38] found extremely preterm more susceptible to catheter-related complications, often leading to repeated catheterizations [46]. The lack of comprehensive safety data for neonatal subgroups is a gap in the literature, especially given historical risks like benzoin tincture on premature skin [47].

The impact of TA on device securement and prevention of failure is inconclusive. A systematic review found that TA may increase failure risk for CVADs post-cardiac surgery in adults compared to sutures [10]. In contrast, an RCT with pediatric PIVCs showed a reduction in failure with TA [22]. Overall, our scoping review suggests that TA is linked to lower complication rates for both CVADs and PIVCs. An adult RCT found lower occlusion rates with TA compared to other securement devices. In pediatrics, an RCT showed reduced unintentional dislodgement and leakage with TA used with an integrated securement dressing (ISD) [22]. For pediatric patients with CVADs, a pilot-RCTs of tunneled catheters found less CLABSI, occlusion, and partial dislodgment in the TA group compared to ISD and suture-less securement device [48], while another pilot-RCT with PICC found no difference in complications between groups [44].

Despite variations in evidence on TA and CVAD complications, systematic reviews support TA’s benefit in prolonging time to the first dressing change in both adults and pediatrics, due to its hemostatic activity [10]. This is crucial for managing immediate post-insertion bleeding and dressing disruption in neonates, which can be linked to catheter-related infections [49]. However, only one study in our scoping review assessed this outcome [35]. Furthermore, increasing catheter dwell time is also important as it reduces insertion procedures, benefiting neonates’ neurodevelopment and long-term vein health [1, 9]. This benefit was observed in both this scoping review and systematic reviews of adults and pediatrics [10].

The scoping review highlights the potential benefits of TA for neonates but notes significant limitations by study design, quality, and bias. Additionally, many studies used TA as part of a bundle, complicating the analysis of TA-specific outcomes. Therefore, RCTs are needed to confirm the effectiveness and safety of TA in neonates, with consideration to nuanced specifics of this population (stratification by gestational age, standardized dose application of TA, and assessing skin injury as a secondary outcome). This would provide much needed data relating to the safety and effectiveness of TA in all neonatal populations, including preterm infants. This scoping review has its own limitations, such as the exclusion of grey literature and the restriction of languages assessed, which may limit the comprehensiveness of the data.

## Conclusion

TA shows promise in enhancing vascular access device securement, potentially reducing complications like dislodgement, CLABSI, and phlebitis. However, evidence for its effectiveness in neonates is limited, as most studies used TA within a bundle of interventions, complicating the assessment of its specific effects. To fill this gap, focused research, particularly RCTs, is needed to evaluate TA’s effectiveness and safety in preventing device failures and complications in neonates. Future studies should also address different subgroups, such as preterm infants, and emphasize safety. High-quality study designs will be essential to validate TA’s impact and improve neonatal care practices.

## Supplementary information

Below is the link to the electronic supplementary material.

Supplementary file1 (DOCX 22 KB)

Supplementary file2 (XLSX 14 KB)

Supplementary file3 (DOCX 30 KB)

## Data Availability

No datasets were generated or analysed during the current study.

## Abbreviations

CICC: Centrally inserted central catheter
CVAD: Central venous access devices
ISD: Integrated securement dressing
FICC: Femorally inserted central catheter
LILACS: Latin American and Caribbean Health Sciences Literature
MMAT: Mixed Methods Appraisal Tool
NBCA: *n*-Butyl-cyanoacrylate
OCA: 2-Octyl-cyanoacrylate
PICC: Peripherally inserted central catheter
PIVC: Peripheral intravenous catheter
PRISMA-ScR: Preferred Reporting Items for Systematic Reviews and Meta-Analyses extension for Scoping Reviews
RCT: Randomized controlled trial
UVC: Umbilical venous catheter
TA: Tissue adhesive
