# Supplementary material for: Use of tissue adhesive for neonatal intravenous access devices: A scoping review
Source: Eur J Pediatr. 2024 Oct 5;183(12):5103–12. doi: 10.1007/s00431-024-05800-3 (PMC11527952; doi:10.1007/s00431-024-05800-3)
Supplement: Supplementary file 1 — Supplementary file1 (DOCX 22 KB) [file 431_2024_5800_MOESM1_ESM.docx]

*European Journal of Pediatrics*

**Use of tissue adhesive for neonatal intravenous access devices: A scoping review**

Sabrina de Souza^1,2*^, Mari Takashima^1,3^, Thiago Lopes Silva^2^, Linda Nugyen^1,3^, Tricia Kleidon^1,3^, Luke Jardine^1,4^, Tim R. Dargavile^5^, Amanda Ullman^1,3^, Patrícia Kuerten Rocha^2†^, Deanne August^1,3†^

^1^The University of Queensland, Brisbane (QLD), Australia; ^2^Universidade Federal de Santa Catarina, Florianopolis (SC), Brazil; ^3^Children’s Health Queensland Hospital and Health Service, Brisbane (QLD), Australia; ^4^Mater Clinic Unit, Brisbane (QLD), Australia; ^5^Queensland University of Technology, Brisbane (QLD), Australia

*Corresponding author. E-mail: [s.desouza@uq.edu.au](mailto:s.desouza@uq.edu.au)

**Supplementary table 1**: Search terms and their combinations for the database search.

| **Search terms** | | |
| --- | --- | --- |
| *Population* | *Intervention* | *Vascular access devices* |
| "Pediatrics"[Mesh]  "Infant"[Mesh]  "Child"[Mesh]  "Adolescent"[Mesh]  pediatric[tiab]  paediatric*child*[tiab]  newborn*[tiab]  new-born*[tiab]  infant* [tiab]  neonat* [tiab] | "Tissue Adhesives"[Mesh] securement[tiab]  cyanoacrylate*[tiab]  superglue*[tiab]  glue[tiab]  "tissue adhesive*"[tiab]) | "Catheterization, Central Venous"[Mesh] "lif"[Mesh]  "Vascular Access Devices"[Mesh]  "peripheral venous cathet*"[tiab]  "non tunnelled"[tiab])  catheterisation[tiab  catheterization[tiab] intravenous[tiab]  "intravenous catheter*"[tiab]  "intravenous line*"[tiab]  tunnelled[tiab]  "peripheral intravenous cannula*"[tiab]  "umbilical catheter*"[tiab]  "umbilical venous catheter*"[tiab]  "long peripheral catheter*"[tiab] midline[tiab]  "tunneled cuffed"[tiab]  "peripherally inserted central catheter*"[tiab]  "peripheral vascular access device*"[tiab]  "vascular access device*"[tiab]  "tunnelled central venous catheter*"[tiab] |
| **Combination** | | |
| **Pubmed**: "peripheral venous cathet*"[tiab] OR catheterisation[tiab] OR catheterization[tiab] OR intravenous[tiab] OR "intravenous catheter*"[tiab] OR "intravenous line*"[tiab] OR tunnelled[tiab] OR "peripheral intravenous cannula*"[tiab] OR "umbilical catheter*"[tiab] OR "umbilical venous catheter*"[tiab] OR "long peripheral catheter*"[tiab] OR midline[tiab] OR "tunneled cuffed"[tiab] OR "peripherally inserted central catheter*"[tiab] OR "peripheral vascular access device*"[tiab] OR "vascular access device*"[tiab] OR "tunnelled central venous catheter*"[tiab] OR "non tunnelled"[tiab]) OR "Catheterization, Central Venous"[Mesh] OR "Catheterization, Peripheral"[Mesh] OR "Vascular Access Devices"[Mesh])  AND  (securement[tiab] OR cyanoacrylate*[tiab] OR superglue*[tiab] OR glue[tiab] OR "tissue adhesive*"[tiab]) OR "Tissue Adhesives"[Mesh])  AND  (pediatric[tiab] OR paediatric*child*[tiab] OR newborn*[tiab] OR new-born*[tiab] OR infant* [tiab] OR neonat* [tiab]) OR "Pediatrics"[Mesh] OR "Infant"[Mesh] OR "Child"[Mesh] OR "Adolescent"[Mesh]) | | |
| **Embase:** ('peripheral venous cathet*':ti,ab OR catheterisation:ti,ab OR catheterization:ti,ab OR intravenous:ti,ab OR 'intravenous catheter*':ti,ab OR 'intravenous line*':ti,ab OR tunnelled:ti,ab OR 'peripheral intravenous cannula*':ti,ab OR 'umbilical catheter*':ti,ab OR 'umbilical venous catheter*':ti,ab OR 'long peripheral catheter*':ti,ab OR midline:ti,ab OR 'tunneled cuffed':ti,ab OR 'peripherally inserted central catheter*':ti,ab OR 'peripheral vascular access device*':ti,ab OR 'vascular access device*':ti,ab OR 'tunnelled central venous catheter*':ti,ab OR 'non tunnelled':ti,ab OR 'central venous catheterization'/exp OR 'catheterization'/exp OR 'vascular access device'/exp OR 'peripheral venous catheter'/exp) AND (securement:ti,ab OR cyanoacrylate*:ti,ab OR superglue*:ti,ab OR glue:ti,ab OR 'tissue adhesive'/exp) AND (pediatric:ti,ab OR paediatric*child*:ti,ab OR newborn*:ti,ab OR 'new born*':ti,ab OR infant*:ti,ab OR neonat*:ti,ab OR 'pediatrics'/exp OR 'infant'/exp OR 'child'/exp OR 'adolescent'/exp) | | |
| **CINAHL:** ( (TI "peripheral venous cathet*" OR AB "peripheral venous cathet*") OR (TI catheterisation OR AB catheterisation) OR (TI catheterization OR AB catheterization) OR (TI intravenous OR AB intravenous) OR (TI "intravenous catheter*" OR AB "intravenous catheter*") OR (TI "intravenous line*" OR AB "intravenous line*") OR (TI tunnelled OR AB tunnelled) OR (TI "peripheral intravenous cannula*" OR AB "peripheral intravenous cannula*") OR (TI "umbilical catheter*" OR AB "umbilical catheter*") OR (TI "umbilical venous catheter*" OR AB "umbilical venous catheter*") OR (TI "long peripheral catheter*" OR AB "long peripheral catheter*") OR (TI midline OR AB midline) OR (TI "tunneled cuffed" OR AB "tunneled cuffed") OR (TI "peripherally inserted central catheter*" OR AB "peripherally inserted central catheter*") OR (TI "peripheral vascular access device*" OR AB "peripheral vascular access device*") OR (TI "vascular access device*" OR AB "vascular access device*") OR (TI "tunnelled central venous catheter*" OR AB "tunnelled central venous catheter*") OR (TI "non tunnelled" OR AB "non tunnelled")OR (MH "Catheterization, Central Venous+") OR (MH "Catheterization, Peripheral+") OR (MH "Vascular Access Devices+") ) AND ( ((TI securement OR AB securement) OR (TI cyanoacrylate* OR AB cyanoacrylate*) OR (TI superglue* OR AB superglue*) OR (TI glue OR AB glue) OR (TI "tissue adhesive*" OR AB "tissue adhesive*")) OR (MH "Tissue Adhesives+") ) AND ( ((TI pediatric OR AB pediatric) OR (TI paediatric*child* OR AB paediatric*child*) OR (TI newborn* OR AB newborn*) OR (TI new-born* OR AB new-born*) OR (TI infant* OR AB infant*) OR (TI neonat* OR AB neonat*)) OR (MH Pediatrics+) OR (MH Infant+) OR (MH Child+) OR (MH Adolescent+) ) | | |
| **Web of science**: "peripheral venous cathet*" OR catheterisation OR catheterization OR intravenous OR "intravenous catheter*" OR "intravenous line*" OR tunnelled OR "peripheral intravenous cannula*" OR "umbilical catheter*" OR "umbilical venous catheter*" OR "long peripheral catheter*" OR midline OR "tunneled cuffed" OR "peripherally inserted central catheter*" OR "peripheral vascular access device*" OR "vascular access device*" OR "tunnelled central venous catheter*" OR "non tunnelled"OR "Catheterization, Central Venous" OR "Catheterization, Peripheral" OR "Vascular Access Devices" AND (securement OR cyanoacrylate* OR superglue* OR glue OR "tissue adhesive*") OR "Tissue Adhesives" AND (pediatric OR paediatric*child* OR newborn* OR new-born* OR infant* OR neonat*) OR Pediatrics OR Infant OR Child OR Adolescent | | |
| **Scopus:** "peripheral venous cathet*" OR catheterisation OR catheterization OR intravenous OR "intravenous catheter*" OR "intravenous line*" OR tunnelled OR "peripheral intravenous cannula*" OR "umbilical catheter*" OR "umbilical venous catheter*" OR "long peripheral catheter*" OR midline OR "tunneled cuffed" OR "peripherally inserted central catheter*" OR "peripheral vascular access device*" OR "vascular access device*" OR "tunnelled central venous catheter*" OR "non tunnelled"OR "Catheterization, Central Venous" OR "Catheterization, Peripheral" OR "Vascular Access Devices" AND (securement OR cyanoacrylate* OR superglue* OR glue OR "tissue adhesive*") OR "Tissue Adhesives" AND (pediatric OR paediatric*child* OR newborn* OR new-born* OR infant* OR neonat*) OR Pediatrics OR Infant OR Child OR Adolescent | | |
| **Cochrane:** ("peripheral venous" NEXT cathet*):ti,ab OR catheterisation:ti,ab OR catheterization:ti,ab OR intravenous:ti,ab OR ("intravenous" NEXT catheter*):ti,ab OR ("intravenous" NEXT line*):ti,ab OR tunnelled:ti,ab OR ("peripheral intravenous" NEXT cannula*):ti,ab OR ("umbilical" NEXT catheter*):ti,ab OR ("umbilical venous" NEXT catheter*):ti,ab OR ("long peripheral" NEXT catheter*):ti,ab OR midline:ti,ab OR "tunneled cuffed":ti,ab OR ("peripherally inserted central" NEXT catheter*):ti,ab OR ("peripheral vascular access" NEXT device*):ti,ab OR ("vascular access" NEXT device*):ti,ab OR ("tunnelled central venous" NEXT catheter*):ti,ab OR "non tunnelled":ti,ab OR [mh "Catheterization, Central Venous"] OR [mh "Catheterization, Peripheral"] OR [mh "Vascular Access Devices"])  AND  (securement:ti,ab OR cyanoacrylate*:ti,ab OR superglue*:ti,ab OR glue:ti,ab OR ("tissue" NEXT adhesive*):ti,ab OR [mh "Tissue Adhesives"])  AND  (pediatric:ti,ab OR paediatric*:ti,ab OR child*:ti,ab OR newborn*:ti,ab OR new-born*:ti,ab OR infant*:ti,ab OR neonat*:ti,ab OR [mh Pediatrics] OR [mh Infant] OR [mh Child] OR [mh Adolescent]) | | |
| **Lilacs:**  (tw:("peripheral venous cathet*") OR tw:catheterisation OR tw:catheterization OR tw:intravenous OR tw:("intravenous catheter*") OR tw:("intravenous line*") OR tw:tunnelled OR tw:("peripheral intravenous cannula*") OR tw:("umbilical catheter*") OR tw:("umbilical venous catheter*") OR tw:("long peripheral catheter*") OR tw:midline OR tw:("tunneled cuffed") OR tw:("peripherally inserted central catheter*") OR tw:("peripheral vascular access device*") OR tw:("vascular access device*") OR tw:("tunnelled central venous catheter*") OR tw:("non tunnelled"))  OR mh:("Cateterismo Venoso Central") OR mh:("Cateterismo Venoso Periférico") OR mh:("Dispositivos de Acesso Vascular"))  AND  ((tw:securement OR tw:cyanoacrylate* OR tw:superglue* OR tw:glue OR tw:("tissue adhesive*")) OR mh:("Adesivos Teciduais"))  AND  ((tw:pediatric OR tw:paediatric* OR tw:child* OR tw:newborn* OR tw:new-born* OR tw:infant* OR tw:neonat*) OR mh:Pediatria OR mh:Infantil OR mh:Criança OR mh:Adolescente) | | |
| **Clinicaltrials.gov:** ("peripheral venous catheter" OR catheterisation OR catheterization OR intravenous OR "intravenous catheter" OR "intravenous line" OR tunnelled OR "peripheral intravenous cannula" OR "umbilical catheter" OR "umbilical venous catheter" OR "long peripheral catheter" OR midline OR "tunneled cuffed" OR "peripherally inserted central catheter" OR "peripheral vascular access device" OR "vascular access device" OR "tunnelled central venous catheter" OR "non tunnelled" OR "Central Venous Catheterization" OR "Peripheral Catheterization" OR "Vascular Access Devices")  AND  (securement OR cyanoacrylate OR superglue OR glue OR "tissue adhesive" OR "Tissue Adhesives")  AND  (pediatric OR paediatric OR child OR newborn OR "new-born" OR infant OR neonate OR Pediatrics OR Infant OR Child OR Adolescent) | | |
| **International Clinical Trial Register Platform:** ("tissue adhesive" OR cyanoacrylate OR glue) AND (children OR pediatric OR neonat* OR infant)  <https://trialsearch.who.int/> | | |
| **Australian and New Zealand Clinical Trials Registry**: ("tissue adhesive" OR cyanoacrylate OR glue) AND (children OR pediatric OR neonat* OR infant) | | |
