# Supplementary material for: Use of tissue adhesive for neonatal intravenous access devices: A scoping review
Source: Eur J Pediatr. 2024 Oct 5;183(12):5103–12. doi: 10.1007/s00431-024-05800-3 (PMC11527952; doi:10.1007/s00431-024-05800-3)
Supplement: Supplementary file 3 — Supplementary file3 (DOCX 30 KB) [file 431_2024_5800_MOESM3_ESM.docx]

*European Journal of Pediatrics*

**Use of tissue adhesive for neonatal intravenous access devices: A scoping review**

Sabrina de Souza^1,2*^, Mari Takashima^1,3^, Thiago Lopes Silva^2^, Linda Nugyen^1,3^, Tricia Kleidon^1,3^, Luke Jardine^1,4^, Tim R. Dargavile^5^, Amanda Ullman^1,3^, Patrícia Kuerten Rocha^2†^, Deanne August^1,3†^

^1^The University of Queensland, Brisbane (QLD), Australia; ^2^Universidade Federal de Santa Catarina, Florianopolis (SC), Brazil; ^3^Children’s Health Queensland Hospital and Health Service, Brisbane (QLD), Australia; ^4^Mater Clinic Unit, Brisbane (QLD), Australia; ^5^Queensland University of Technology, Brisbane (QLD), Australia

*Corresponding author. E-mail: [s.desouza@uq.edu.au](mailto:s.desouza@uq.edu.au)

**Supplementary table 3:** Description of the studies and outcomes

| **STUDIES ASSESSING SPECIFICALLY TA** | | | | | | | | | | | |
| --- | --- | --- | --- | --- | --- | --- | --- | --- | --- | --- | --- |
| **Author** | **Study design** | **Sample size** | **Vascular access device using TA** | **Vein/ location of the vein** |  | | | | | | |
|  |  |  |  |  | **Failure** | | **Complications** | | | **Catheter dwell time** | |
| D’Andrea et al. [37] | RCT | 130 neonates included/ 130 catheters | UVC | Umbilical vein | Control group  (**non-TA**)  3(4.6%) | Intervention group (**TA**)  4(6.2%) |  | **Non-TA** | **TA** | N/I | |
|  |  |  |  |  |  |  | Overall dislodgment | 16 (24.6%) | 5 (7.7%)  **p-value=0.016** |  |  |
|  |  |  |  |  |  |  | Migration without dislodgment | 6 (12.2%) | 7 (11.7%) |  |  |
|  |  |  |  |  |  |  | CLABSI | 5 (7.7%) | 2 (3.1%) |  |  |
|  |  |  |  |  |  |  | UVC-related thrombosis | 5 (7.7%) | 4 (6.1%) |  |  |
|  |  |  |  |  |  |  | Overall dislodgment for neonates < 1500g | 32.4% | 8.3%  **p < 0.03** |  |  |
| vanRens et al [38] | Observational study (retrospective) | 1842 neonates and catheters | PICC | Upper or lower extremity veins | **Non-TA:**  n= 259 (27.9%) | **TA:**  n = 91 (11.7%)  **p-value < 0.001** |  | **Non-TA** | **TA** | **Non-TA** (days)  11 (7-17) | **TA**  (days)  10 (7-14)  p-value= 0.074 |
|  |  |  |  |  |  |  | Leaking or breakage | 24 (2.5%) | 19 (2.2%) |  |  |
|  |  |  |  |  |  |  | Extravasation/ infiltration | 47 (4.8%) | 3 (0.3%) |  |  |
|  |  |  |  |  |  |  | Accidental removal and occlusion | 53 (4%) | 22 (2.5%) |  |  |
|  |  |  |  |  |  |  | Phlebitis | 42 (4.3%) | 14 (1.6%) |  |  |
|  |  |  |  |  |  |  | Suspected sepsis | 87 (8.9%) | 31 (3.6%) |  |  |
|  |  |  |  |  |  |  | Death (CLABSI) | 6 (0.6%) | 2 (0.2%) |  |  |
|  |  |  |  |  |  |  | CLABSI incidence | 2.76/1000 days | 0.99/1000 days  **(p<0.001)** |  |  |
| vanRens et al [24]* | Observational (retrospective) | 12978 neonates/ 15087 catheters | PIVC | Hand, Wrist/lower arm, Elbow/upper arm, foot, ankle/lower leg, knee/upper leg, Scalp | 7627 participants (59%). | | The overall complication rate was 18 per 1000 catheters days.  Infiltration/extravasation: 5159 (40%)  Phlebitis 1590 (12%)  Occlusion 527 (4%)  Dislodgment/accidental removal 286 (2%)  Swelling or discoloration 65 (1%) | | | **Non-TA**  (hours)  28 ± 18 | **TA**  (hours)  34 ± 25 |
| vanRens et al [39] | Observational (retrospective) | 8830 catheters and neonates | PIVC | Elbow, foot, hand, lower arm, lower leg, upper arm, and upper leg | **Non-TA**  2753 (63%) | **TA**  1895 (51%)  Odds ratio 0.59 (0.54-0.65) **p-value < 0.001** |  | **Non-TA** | **TA** | **Non-TA**  (Hours)  31 ± 24.3 | **TA**  (hours)  37.1 ± 31.1 |
|  |  |  |  |  |  |  | Accidental dislodgment | 130 (3%) | 105 (3%) |  |  |
|  |  |  |  |  |  |  | Leaking | 445 (10%) | 386 (10%) |  |  |
|  |  |  |  |  |  |  | Occlusion | 219 (5%) | 160 (4%) |  |  |
|  |  |  |  |  |  |  | Phlebitis | 594 (13%) | 123 (3%) |  |  |
|  |  |  |  |  |  |  | Infiltration/extravasation | 1365 (31%) | 1121 (29%) |  |  |
| D’Andrea et al [36] | Quasi-experimental | 172 neonates and 134 catheters | PICC | Antecubital fossa, saphenous, popliteal vein, scalp | Control group  (**Non-TA**):  35 (28.2%) catheters | Intervention (**TA**):  39 (29.1%) catheters |  | **Non-TA** | **TA** | **Non-TA** | **TA** |
|  |  |  |  |  |  |  | Dislodgment | 14 (11.3%) | 1 (0.7%)  **p-value= 0.0003** | (days)  11 | (days) 10.4 |
| **STUDIES ASSESSING A BUNDLE** | | | | | | | | | | | |
| **Author** | **Study design** | **Sample size** | **Vascular access device using TA** | **Vein/ location of the vein** | **BUNDLE OUTCOME** | | | | | | |
|  |  |  |  |  | **Failure** | | **Complications** | | | **Catheter dwell time** | |
| Spagnuolo et al [34] | Observational (prospective) | 104 neonates and catheters | CVAD:  CICC and FICC | Supra-infraclavicular region, for CICC and lower extremities for FICC | N/I | | CRBSI was 2.47—1000 catheter days.  Post-procedural complications reported in the first 2 weeks:  Dislodgment (n=1, 0.96%)  Secondary misplacement (n=1, 0.96%)  Infection of the exit site (n=1, 0.96%) | | | 12 days (±2–90). | |
| Bierlaire et al. [33] | Quasi-experimental | 430 neonates and 417 catheters | CVAD: CICC and PICC | N/I | N/I | |  | Pre-intervention (non-TA) | Post intervention  (TA) | N/I | |
|  |  |  |  |  |  |  | CLABSI total | 18 | 3  **p-value= 0.04** |  |  |
|  |  |  |  |  |  |  | CLABSI in neonates < 32 weeks | 16 | 1  **p-value= 0.01** |  |  |
|  |  |  |  |  |  |  | Mechanical complications (broken, occluded, disconnected) | 13 (5%) | 1 (0.6%)  **p-value=** **0.015** |  |  |
|  |  |  |  |  |  |  | Local concerns (erythema, phlebitis, extravasation) | 8 (3%) | 4 (2.5%)  p-value= 0.73 |  |  |
|  |  |  |  |  |  |  | Dislocation/migration | 21 (8%) | 3 (1.9%)  **p-value=** **0.007** |  |  |
|  |  |  |  |  |  |  | Other (Pleural effusion, arrythmia, deep venous thrombosis) | 4 (2%) | 2 (1.2%)  p-value= 0.8 |  |  |
| D’Andrea et al [32] | Case series | 70 neonates and 72 catheters | CVADs: CICC and FICC | Brachio-cephalic vein and Femoral vein | None | | None | | | 39 (± 25) days | |
| Pittiruti et al [31] | Observational (prospective) | 60 neonates and catheters | CVADs: FICC and CICC | Brachiocephalic, internal jugular, external jugular, axillary, femoral | None | | None | | | N/I | |
| Spagnuolo et al [30] | Observational (retrospective) | 40 neonates and catheters | CVAD: CICC | Brachiocephalic vein | Two catheters had to be removed prematurely due to CRBSI 16 (5%) | | CRBSI: 4.5 infections per 1000 catheter days  No catheter related thrombosis. | | | 10.88 ± 6.41 (4-29) days. | |
| Barone et al [29] | Case series | 30 neonates and catheters | CVAD: CICC | Brachio-cephalic vein/ infraclavicular | None | | None | | | 37 ± 21 (2-95) days | |
| Piersigilli et al [35] | Quasi-experimental | 350 neonates and 428 catheters | PICC | N/I | N/I | | Total number of complications: 20 (pre-intervention), 8 (post-intervention)  Dislodgement: reduction in 13 to 1 post implementation (**p-value <0.0001**).    CLABSI: reduction 12 episodes vs 5 episodes with a decrease from 9.8 episodes/1000 line days to 1.4/1000 line days (**p-value <0.0001**).  Thrombosis and occlusion reduction were not statistically significant. | | | Increase of 9 to 11 days | |
